# A unified framework for focal intensity change detection and deformable image registration. Application to the monitoring of multiple sclerosis lesions in longitudinal 3D brain MRI

**DOI:** 10.3389/fnimg.2022.1008128

**Published:** 2022-12-22

**Authors:** Eléonore Dufresne, Denis Fortun, Stéphane Kremer, Vincent Noblet

**Affiliations:** ^1^ICube UMR 7357, Université de Strasbourg, CNRS, Strasbourg, France; ^2^Hôpitaux Universitaires de Strasbourg, Strasbourg, France

**Keywords:** deformable 3D registration, change detection, longitudinal analysis, multiple sclerosis, joint minimization, alternating direction method of multipliers (ADMM)

## Abstract

Registration is a crucial step in the design of automatic change detection methods dedicated to longitudinal brain MRI. Even small registration inaccuracies can significantly deteriorate the detection performance by introducing numerous spurious detections. Rigid or affine registration are usually considered to align baseline and follow-up scans, as a pre-processing step before applying a change detection method. In the context of multiple sclerosis, using deformable registration can be required to capture the complex deformations due to brain atrophy. However, non-rigid registration can alter the shape of appearing and evolving lesions while minimizing the dissimilarity between the two images. To overcome this issue, we consider registration and change detection as intertwined problems that should be solved jointly. To this end, we formulate these two separate tasks as a single optimization problem involving a unique energy that models their coupling. We focus on intensity-based change detection and registration, but the approach is versatile and could be extended to other modeling choices. We show experimentally on synthetic and real data that the proposed joint approach overcomes the limitations of the sequential scheme.

## 1. Introduction

Multiple sclerosis (MS) is an auto-immune neurodegenerative disease characterized by the inflammation of the myelin coating that surrounds the nerves. As a consequence, the transmission of nervous impulses is impaired, causing motor, cognitive and sensorial disabilities. The evolution of MS is characterized by the apparition of focal lesions in the brain and in the spinal cord, and by a progressive atrophy of brain tissues. Both phenomena can be monitored thanks to Magnetic Resonance Imaging (MRI) (Kaunzner and Gauthier, 2017). In the clinical routine, the evolution of the lesion load and of the brain atrophy is generally assessed qualitatively. However, the precise quantification of lesion changes over time may be of great interest to finely characterize the course of the pathology and to evaluate at the early stage the effect of a therapeutic strategy (McNamara et al., 2017). Since the manual delineation of lesion changes in MRI is a tedious and time consuming task, which is prone to both intra- and inter-observer variability, there is a great need for efficient and reliable automated tools (Altay et al., 2013).

Most change detection methods dedicated to lesion monitoring rely on a sequential scheme that first consists in removing all changes that are not of interest in order to detect in a second step only the evolution of lesions (Radke et al., 2005). To correct for global intensity changes induced by the difference of MRI acquisition setups, algorithms for bias field inhomogeneity correction (Song et al., 2017) and histogram-based intensity normalization procedure (Shinohara et al., 2014) are generally considered. Then, geometrical discrepancies due to variation in patient positioning, acquisition-related geometrical distortion and brain atrophy are corrected thanks to registration algorithms involving either rigid, affine or deformable transformations. Finally, the remaining changes corresponding to the evolution of lesions are detected. This final step generally consists in thresholding an intensity-based (Bosc et al., 2003) or deformation-based (Rey et al., 2002) feature map. The threshold can be chosen according to some statistical modeling in order to control the expected number of false positive detections (Rousseau et al., 2007).

The main flaw of the sequential procedure is that it implicitly assumes that each correction step can be performed while not being influenced by the changes that remain to be corrected in the next steps. As a consequence, the sequence order of the correction procedures should be carefully chosen. Moreover, for each correction step, a trade-off should be found between its performance (i.e., the ability of the method to accurately and specifically correct a given kind of change) and its robustness (i.e., the ability not to be biased by another kind of remaining changes). This observation advocates for a unified formulation of the change detection problem allowing to estimate all the different kinds of changes jointly.

In this paper, we address more specifically the interplay between deformable image registration and focal intensity change detection. When deformable registration is performed in the presence of appearing lesions, the estimated transformation tends to make these new lesions disappear in order to minimize the dissimilarity between the two images. This is the reason why the most common practice is to consider only rigid or affine registration in order not to alter lesion shape. However, such linear transforms can only compensate for difference in patient positioning but are not able to capture the complex deformations induced by brain atrophy, which typically occurs in MS. These remaining deformations may yield to spurious detections in atrophied areas, especially in the cortex and around the ventricles.

We propose to account for the intertwining of deformable registration and focal intensity change detection by estimating them jointly. To this end, we show that these two separate tasks can be formulated as a single optimization problem involving a unique energy that models their coupling. Basically, areas corresponding to detected changes are ignored in the registration similarity criterion, which prevents the lesion elimination effect described above. Solving this issue allows us to use of deformable registration, which in turn prevents from detecting spurious changes in atrophied areas. We propose an efficient alternating optimization scheme to solve this unified optimization problem. We focus on demonstrating the benefits of this joint formulation in the particular case of a standard intensity-based data similarity criterion. Nevertheless, the proposed approach is versatile and could easily be extended to more elaborated modeling choices. Experimental analysis is performed on the BrainWeb synthetic dataset and on two annotated real datasets. We first demonstrate in each case the benefits of considering a deformable registration as compared to an affine registration only in order to reduce the number of false detections. Then, we highlight the benefit of the proposed joint formulation as compared to the standard sequential scheme in terms of change detection accuracy. A preliminary version of this work has been published as a conference paper in Dufresne et al. (2020). In this paper, we provide a more extensive experimental analysis, which helps to better characterize the behavior of the proposed method and better understand why it outperforms the sequential approach.

The paper is organized as follows. In Section 2, the sequential approach, which will be considered as the reference baseline method of this work, is described and its limitations are discussed. In Section 3, we introduce the proposed joint model and the optmization strategies that has been set up estimate both change detection map and deformation field. In Section 4, we give implementation details. Finally, we present and discuss experimental results in Section 5.2.

## 2. The sequential approach

The conventional sequential approach consists of three main steps. First, the images are corrected for global intensity variations, then they are spatially registered, and finally the focal intensity changes due to the evolution of lesions are detected. In this section, we give a brief overview of the common practices in the registration and change detection steps. Our goal is not to cover a comprehensive scope of the field but to formulate the general principles underlying existing methods. In the remainder of this article, we will denote *I*_1_, *I*_2_: Ω → ℝ the baseline and follow-up MRI images, where Ω ⊂ℝ^3^ is the image domain.

### 2.1. Registration

The registration problem can be formulated as:


(1) $$\begin{array}{l} \hat{\text{w}}=\underset{\text{w}}{\text{argmin}}\;\underset{\text{x}\in \Omega }{\sum }\rho \left(I_{1},I_{2},\text{w},\text{x}\right)+\lambda_{1}\text{Ψ}\left(\text{w}\right), \end{array}$$

where w: Ω → ℝ^3^ represents the transformation, ρ(·) is a data similarity term, and Ψ(·) is a regularizer weighted by a scalar λ_1_ > 0. An overview of deformable registration methods in medical imaging can be found in Sotiras et al. (2013).

Several transformation models can be considered relying either on a parametric representation (e.g., rigid, affine, polynomial, or Bspline-based) or on a non-parametric deformable mapping (i.e., a displacement vector is estimated for each voxel).

The role of the data term is to penalize dissimilarity between *I*_1_ and *I*_2_ warped with the estimated transformation. For monomodal registration, it is common to use intensity-based measures such as the sum of squared intensity differences or the cross correlation. In the multimodal case, mutual information is one of the most widely used similarity metric (Kaunzner and Gauthier, 2017).

In the context of deformable registration, considering the data term only can lead to an ill-posed problem. To overcome this issue, the data term has to be balanced with an additional regularization term Ψ(w) that enforces some constraints on the deformation field. For instance, penalizing the ℓ_2_ or ℓ_1_ norm of the gradient of w helps to promote smooth solutions.

### 2.2. Change detection

The change detection step generally consists in thresholding a map of feature differences between registered baseline and follow-up images (see the survey Lladó et al., 2012). These maps can be calculated directly from either intensity-based (Sweeney et al., 2013; Ganiler et al., 2014; Cabezas et al., 2016), or deformation-based (Rey et al., 2002; Cabezas et al., 2016; Salem et al., 2018) features, and sometimes integrate other kind of information (Elliott et al., 2013; Sweeney et al., 2013).

In the perspective of integrating both registration and change detection in a single joint optimization problem, we advocate that they should both rely on the same data similarity. Consequently, we model the binary change map *c*: Ω → {0, 1} defined at each voxel x as follow:


(2) $$\begin{array}{l} c\left(\text{x}\right)=\{\begin{array}{cc} 0 & \text{if}\rho \left(I_{1},I_{2},\text{w},\text{x}\right)\leq \lambda_{2}\\ 1 & \text{otherwise}, \end{array} \end{array}$$

$\lambda_{2}\in {\mathbb{R}}^{+*}$ being the detection threshold. The thresholding scheme (Equation 2) can be reformulated as the following optimization problem:


(3) $$\begin{array}{l} \hat{c}\left(\text{x}\right)=\underset{c:\Omega \to \left\{0,1\right\}}{\text{argmin}}\underset{\text{x}\in \Omega }{\sum }\left(1-c\left(\text{x}\right)\right)\;\rho \left(I_{1},I_{2},\text{w},\text{x}\right)+\lambda_{2}c\left(\text{x}\right). \end{array}$$

Since simple thresholding can yield noisy results, most MS lesion change detection methods also integrate a denoising step in post-processing to obtain the final change map. The denoising can be realized jointly with the change detection by integrating a regularization term Φ(·) in Equation (3):


(4) $$\begin{array}{l} \hat{c}\left(\text{x}\right)=\underset{c:\Omega \to \left\{0,1\right\}}{\text{argmin}}\underset{\text{x}\in \Omega }{\sum }\left(1-c\left(\text{x}\right)\right)\;\rho \left(I_{1},I_{2},\text{w},\text{x}\right)+\lambda_{2}c\left(\text{x}\right)+\lambda_{3}\Phi \left(c\right), \end{array}$$

Where λ_3_ weights the regularization term.

### 2.3. Limitation of the sequential approach

**Figure 1:**
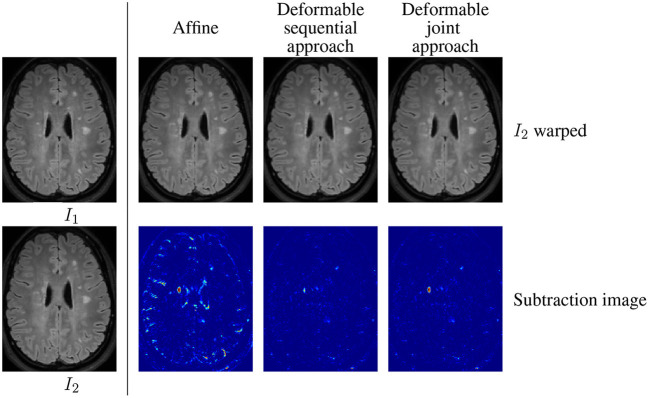
Warped follow-up images and subtraction images obtained with the sequential affine and deformable registration pipelines and the proposed joint deformable approach, on an example of the dataset described in COM (2021).

The main limitation of the sequential approach is illustrated in Figure 1 involving the baseline (Figure 1) and follow-up (Figure 1) MRI acquisitions of a patient suffering from MS. One can observe in the follow-up scan the apparition of a new lesion and a slight enlargement of the ventricle reflecting the brain atrophy process. In the case of affine registration (Figures 1), we can see on the subtraction image that the lesion is well detected, but that spurious detection occur around the ventricles and in the cortical regions due to brain tissue atrophy. Using a deformable registration (Figures 1) helps to remove these spurious detection by compensating the ventricles enlargement and the cortical atrophy. However, it also tends to make the new lesion disappear (Figure 1), thus altering the shape of the corresponding detection (Figure 1). The goal of the proposed joint approach (Figures 1) is to perform an accurate atrophy correction while preserving the shape of appearing and evolving lesions, even when the lesion-to-tissue contrast is quite low.

## 3. Joint approach

### 3.1. General formulation

To overcome the limitations of the sequential approach, we advocate a joint modeling of registration and change detection. The two steps are fundamentally intertwined, since registration aims at finding correspondences between images, while change detection determines regions that does not admit correspondences. Therefore, both tasks should be defined with the same objective function to work in synergy. We formulate the following joint minimization problem that achieves this goal by unifying the principles described previously:


(5) $$\begin{array}{l} \begin{array}{c} \hat{\text{w}},\hat{c}=\underset{\text{w},c}{\text{argmin}}\underset{\text{x}\in \Omega }{\sum }\left[\left(1-c\left(\text{x}\right)\right)\;\rho \left(I_{1},I_{2},\text{w},\text{x}\right)+\lambda_{2}c\left(\text{x}\right)\right]\\ \;+\lambda_{1}\text{Ψ}\left(\text{w}\right)+\lambda_{3}\Phi \left(c\right). \end{array} \end{array}$$

With this model, the data term is cancelled in change regions (where *c*(x) = 1), so that the estimation of the transformation is only driven by the regularization term, thus producing smoothed deformation field in these areas. By this way, it prevents the lesion disappearing effect observed in Figure 1.

### 3.2. Modeling choices

The formulation (Equation 5) is versatile and could be instantiated with a variety of data and regularization terms. In this paper, the goal is to demonstrate the superiority of the joint formulation over the sequential approach under standard modeling choices, which are detailed in the sequel.

First, we assume that, thanks to the intensity normalization step done as a preprocessing, intensities of both images are comparable, thus allowing us to consider a data term based on intensity difference. Consequently, we consider the following standard similarity measure:


(6) $$\begin{array}{l} \rho \left(I_{1},I_{2},\text{w},\text{x}\right)=\frac{1}{\sigma^{2}}||I_{2}\left(\text{x}-\text{w}\left(\text{x}\right)\right)-I_{1}\left(\text{x}\right){||}_{2}^{2}, \end{array}$$

Where σ is a normalization constant defined by the median absolute deviation of the intensity differences between *I*_1_ and *I*_2_. This data term has been used for motion estimation (Bruhn et al., 2005) and is representative of intensity-based features commonly used in change detection methods (Sweeney et al., 2013; Ganiler et al., 2014).

Secondly, we assume that the deformations induced by brain tissue atrophy are complex but still locally smooth. This is why we consider a non-parametric representation of the transformation field w while introducing a first order Tikhonov regularization term:


(7) $$\begin{array}{l} \text{Ψ}\left(\text{w}\right)=\underset{\text{x}\in \Omega }{\sum }||\nabla \text{w}\left(\text{x}\right){||}_{2}^{2}, \end{array}$$

Where ∇· is the gradient operator.

Finally, we assume that the detected changes should be spatially coherent. Consequently, the change map *c* is regularized with a standard binary Potts model:


(8) $$\begin{array}{l} \Phi \left(c\right)=\underset{\text{x}\in \Omega }{\sum }\underset{\text{y}\in N\left(\text{x}\right)}{\sum }\left(1-\delta \left(c\left(\text{x}\right),c\left(\text{y}\right)\right)\right), \end{array}$$

Where δ is the Kronecker function equal to 1 if its argument is true and N(x) is the 6-neighborhood of x.

### 3.3. Optimization

To solve the optimization problem (Equation 5), we rely on an alternating minimization strategy: at each iteration, we successively minimize with respect to (w.r.t.) each variable, while keeping the other fixed. We detail in this section the optimization strategies dedicated to each of the two subproblems.

Notice that, since the problem is nonconvex, convergence toward the global minimum cannot unfortunately be guaranteed. However, the results presented in Section 5.2 on several datasets suggest that the optimization process converges in practice toward a satisfying solution.

**Minimization w.r.t**. **w** To make the problem tractable, we consider the linearized version of the data term (Equation 6) obtained by replacing *I*_2_(y − w(x)) with its Taylor expansion around y:


(9) $$\begin{array}{l} \rho_{l}\left(I_{1},I_{2},\text{w},\text{x}\right)=\frac{1}{\sigma^{2}}||\nabla^{⊤}I_{2}\left(\text{x}\right)\text{w}\left(\text{x}\right)+I_{t}\left(\text{x}\right){||}_{2}^{2}, \end{array}$$

Where *I*_*t*_(y) = *I*_2_(y)−*I*_1_(y) is the temporal derivative. Since the Taylor expansion is valid only for small deformations, we embed the estimation in a coarse-to-fine scheme, which is a common practice in registration and motion estimation (Hill et al., 2001).

After Taylor development of *I*_2_(y − w(x)), the new data term ρ_*l*_ in Equation (9) is the composition of a quadratic function with a linear function of w, which yields a convex term. Since the regularization term (Equation 7) is also convex, the whole optimization problem is convex. By substituting ρ by ρ_*l*_ in Equation (5), the optimisation problem can be addressed with a variety of efficient optimization methods.

We chose to consider the alternated direction method of multipliers (ADMM) framework (Boyd et al., 2011). To this end, we introduce a splitting variable z that decouples the two terms of Equation (5) that depend on w, and we formulate the problem in the constrained form:


(10) $$\begin{array}{l} \underset{\text{w}}{\text{min}}\;\underset{x\in \Omega }{\sum }\left(1-c\left(\text{x}\right)\right)\;\rho_{l}\left(I_{1},I_{2},\text{w},\text{x}\right)+\lambda_{1}\text{Ψ}\left(\text{z}\right)\;\text{s.t w}=\text{z} \end{array}$$

The ADMM algorithm is based on the minimization of the augmented Lagrangian associated with Equation (10) w.r.t. w and z, and a gradient ascent on the dual variable (Boyd et al., 2011). It leads to the following iterative updates of w and z (see Fortun et al., 2018 for a similar derivation with different data and regularization terms):


(11) $$\begin{array}{l} {\text{w}}^{k+1}={\text{prox}}_{\underset{x}{\sum }\left(1-c\left(\text{x}\right)\right)\rho_{l}\left(I_{1},I_{2},\cdot ,\text{x}\right)}\left({\text{z}}^{k}-\frac{\alpha^{k}}{\mu }\right) \end{array}$$

(12) $$\begin{array}{l} {\text{z}}^{k+1}={\text{prox}}_{\lambda_{1}\text{Ψ}}\left({\text{w}}^{k+1}+\frac{\alpha^{k}}{\mu }\right) \end{array}$$

(13) $$\begin{array}{l} \alpha^{k+1}=\alpha^{k}+\mu \left({\text{w}}^{k+1}-{\text{z}}^{k+1}\right) \end{array}$$

Where ${\text{prox}}_{f}\left(\text{x}\right)=\underset{\text{y}}{\text{argmin}}\frac{1}{2}||\text{x}-\text{y}{||}_{2}^{2}+f\left(\text{y}\right)$ denotes the proximity operator of a function *f*. The subproblem (Equation 11) is voxel-wise and quadratic, and it admits a simple closed form solution. The subproblem (Equation 12) is equivalent to a denoising operation with the regularizer Ψ(·), and it also has a closed form linear solution that can be computed efficiently in the Fourier domain. *μ* is the parameter associated with the quadratic penalty in the Augmented Lagrangian associated with Equation (10). The ADMM algorithm is derived from this Augmented Lagrangian and the update (Equations 11, 12) are its minimization w.r.t. w and z. Intuitively, *μ* controls how fast the constraint w = z is imposed through the optimization process. Thus, even if it is not strictly speaking a step size, it has a similar impact on the convergence speed.

Notice that the ADMM framework is flexible enough to cope with different data and regularization terms with low computational cost. The requirement is to be able to design a splitting of the cost function such that the proximity operators of each iteration have computationally efficient solutions. Examples of admissible models comprise data terms based on the ℓ_1_ penalty function or cross-correlation (Vogel et al., 2013), and regularizations by total variation or Nuclear norm of the Jacobian (Bostan et al., 2014).

**Minimization w.r.t**. **c** When w is fixed, the estimation of *c* amounts to a binary segmentation problem with Potts regularization:


(14) $$\begin{array}{l} \begin{array}{c} \hat{c}=\underset{c}{\text{argmin}}\underset{\text{x}\in \Omega }{\sum }\left[\lambda_{2}-\;\rho \left(I_{1},I_{2},\text{w},\text{x}\right)\right]c\left(\text{x}\right)+\lambda_{3}\Phi \left(c\right). \end{array} \end{array}$$

We solve it with a graph-cut method (Boykov et al., 2001), which is able to find an exact solution with very low computational cost.

## 4. Implementation details

### 4.1. Pre-processing

Before applying the change detection framework, the input images require to be pre-processed as follows. First, images are corrected for bias field inhomogeneity using the N4 algorithm (Tustison et al., 2010). Then, a global scaling of the intensity is performed in order to enforce the median value of the intensities inside the brainmask to be equal to 100. Images are then resampled to 1 mm isotropic resolution. These two steps aim at harmonizing all input images in terms of intensity range and spatial resolution. The follow-up scan is then rigidly registered on the baseline image using ANTs library (Avants et al., 2011)^1^ with default parameters and mutual information metric. Differential bias field inhomogeneity is corrected thanks to the method described in Lewis and Fox (2004), while considering a 21 × 21 × 21 median filter size.

### 4.2. Post-processing

The detection maps are post-processed by discarding the connected components smaller than 3mm^3^ and the detections outside brain parenchyma (i.e., the union of gray and white matter). Notice that the binary change detection map can also be computed to reflect either positive or negative intensity changes only.

Brain parenchyma masks are computed from T1-weighted images using the FAST method provided in the FSL library (Zhang et al., 2001) or alternatively from FLAIR images using SAMSEG^2^ brain parcellation tool (Cerri et al., 2021), in the case where no T1-weighted image is available.

### 4.3. Hyperparameter setting

Three hyperparameters have to be set in the proposed joint formulation (Equation 5): λ_1_, controlling the spatial regularization of the deformation field, λ_2_ acting as a threshold for the map of intensity differences, and λ_3_, controlling the spatial regularization of the change map. Here, we suggest strategies to find out relevant parameters settings.

The value of λ_1_ should be chosen to optimally estimate longitudinal brain atrophy, since it is the main source of brain deformation in MS. Thus, we consider a subset of 21 images from the dataset OASIS-3 (LaMontagne et al., 2019) that contains longitudinal Alzheimer and normal aging MRI data that exhibit various pattern of longitudinal brain atrophy. We determine the optimal value of λ_1_ by selecting the one that leads to the best registration performance on this subset of OASIS-3. To this end, we derive a registration quality metric from the provided segmentation maps of brain structures obtained with Freesurfer. Concretely, the Dice score is computed for each structure between the segmentation maps of the baseline image and of the registered follow-up image. The global registration quality metric is then computed as the sum over all the regions of the median Dice score observed for each region. This procedure leads us to find λ_1_ = 70 as an optimal value.

The values of λ_2_ and λ_3_ have to be set to find the best compromise regarding: (i) The expected intensity difference, (ii) the noise level that corrupts the images, and (iii) the spatial extent of the changes. Here, we suggest an approach to find out optimized setting for each of the two considered databases (see Sections 5.1.2, 3.2). In practice, λ_2_ and λ_3_ have been fixed to maximize the overall performance of the *affine sequential* approach (see Section 5.1.5) in terms of local Dice Similarity Coefficient (*local* DSC, see Section 5.1.4) for each dataset. Considering the *local* DSC ensures to focus on the ability of the detection scheme (Equation 14) to recover the detected changes while not being influenced by false positive detections that can occur in other parts of the brain. Considering the affine transformation model ensures that the registration step does not to alter the geometry of evolving regions. This procedure leads us to find λ_2_ = 16 and λ_3_ = 5 as optimal setting for LesjakDB dataset and λ_2_ = 25 and λ_3_ = 3 for MSSEG-2 dataset.

### 4.4. Convergence and stopping criteria

The iterations of the alternated minimization of Equation (5) and of the ADMM algorithm (Equations 11–13) are stopped when a stopping criterion is verified or when a maximum number of iterations is reached. The stopping criterion is a threshold on the norm of the relative changes between two consecutive iterations, and is set to 10^−3^ for the alternated minimization and 2.10^−3^ for ADMM. The maximum number of iterations is set to 5 for the alternated minimization and 300 for ADMM.

## 5. Experimental evaluation

### 5.1. Evaluation framework

In this section, we report results obtained on one synthetic dataset and two publicly available real patients datasets. The synthetic dataset offer the advantage to have an unambiguously defined ground truth change detection map, while controlling the amount of noise, bias field inhomogeneity and brain atrophy that corrupt the images. The real datasets are used to evaluate the proposed approach in conditions that are closer to the clinical routine, while considering different acquisition conditions and various pathological evolution. The first real patients dataset, denoted in the sequel as *LesjakDB* (Lesjak et al., 2016), is dedicated to assess the ability of methods to detect every kinds of MS lesion evolutions (shrinkage, growth, new and disappearing), whereas the second dataset, denoted in the sequel as *MSSEG-2* (COM, 2021), only focus on the ability to detect new appearing lesions.

#### 5.1.1. Synthetic dataset

We evaluate the proposed method on T2-weighted synthetic volumes generated with the Brainweb simulator (Cocosco et al., 1997), while considering the *normal* anatomical model (i.e., without lesion) and two multiple sclerosis anatomical models with *moderate* and *severe* lesion load. The images are simulated at a 1 *mm*^3^ isotropic resolution (image size: 181 x 217 x 181) with bias field inhomogeneity (20%). To simulate realistic brain atrophy for the follow-up image, we applied a deformation field that has been estimated using a deformable registration (Avants et al., 2008) from two T1-weighted MRI scans acquired 4 years apart of a patient suffering from MS that exhibits a significant brain atrophy evolution (in-house dataset). It should be noted that these real data images were first affinely registered onto the brainweb image to ensure the estimated deformable registration to be consistent with the underlying anatomy. The visual inspection confirms that the simulated image exhibits a realistic atrophy pattern. Gaussian additive noise was added with a standard deviation fixed at 5% of the mean intensity in the brightest tissue (cerebrospinal fluid in the T2-weighted simulation). We consider several scenarios of simulated longitudinal acquisition that are summarized in Table 1.

**Table 1:** Simulated longitudinal acquisitions.

**Scenario**	**Baseline image**	**Follow-up image**	**Simulated atrophy**
Lesion appearance without atrophy	Normal	Moderate	No
Lesion growth without atrophy	Moderate	Severe	No
Lesion appearance with atrophy	Normal	Moderate	Yes
Lesion growth with atrophy	Moderate	Severe	Yes

#### 5.1.2. Real dataset LesjakDB: All kinds of lesion evolution

LesjakDB dataset (Lesjak et al., 2016) is composed of 20 longitudinal MRI acquisitions of MS patients with two timepoints. The median time between the baseline and follow-up studies was 311 days, ranging from 81 to 723 days. Each MRI acquisition consists in a 2D T1-weighted, a 2D T2-weighted and 2D-FLAIR sequences. Change detection was conducted on the FLAIR images only. The FLAIR image size is 256 × 256 × 49 with an anisotropic spatial resolution of 0.9 × 0.9 × 3 mm. Ground truth change detection maps are also provided, which were obtained from manual annotations done by two expert raters. We adjusted some of the ground truth annotations that did not match the real lesion changes. The annotated changes include appearing, growing, shrinking and disappearing lesions. Ground-truth detection maps are compared to binary detection maps that include both positive and negative intensity changes.

#### 5.1.3. Real dataset MSSEG-2: Only appearing lesions

MSSEG-2 dataset (COM, 2021) is composed of 100 pairs of FLAIR MRI scans from MS patients acquired on various MR scanners. The provided ground-truth is limited to new appearing lesions, and was build from the consensus of manual annotations delineated by four experts. The dataset is separated into training (40 patients) and testing (60 patients) sets. Since the proposed approach does not require any training step, we consider the whole dataset for testing. However, we distinguish two subgroups of data, namely *MSSEG-2-Change* corresponding the 61 subjects that exhibit at least one new appearing lesion and *MSSEG-2-NoChange* corresponding the 39 subjects that do not exhibit any new appearing lesion. Since the provided ground-truth is limited to new appearing lesions, they are compared only to the positive binary change detection maps obtained with the different methods. Notice, that the proposed method framework does not discriminate appearing from evolving lesion. Consequently, lesion evolutions, which are not labeled in the ground truth detection maps, are erroneously considered as false positive detection, thus introducing a bias in some of the evaluation metrics.

#### 5.1.4. Metrics

We report four metrics to evaluate the performance of the methods to detect changes, namely the Dice Similarity Coefficient (DSC), the Positive Predictive Value (PPV), the True Positive Ratio (TPR) and the *local* DSC. Let *TP*, *TN*, *FP*, and *FN* be the number of voxels from estimated change detection map that correspond to *True Positive, True Negative, False Positive* and *False Negative*, respectively.

The DSC is defined as:


$$\begin{array}{l} DSC=2TP/\left(2TP+FP+FN\right) \end{array}$$

and reflects the overall good overlap between the detection map and the ground truth.

The PPV is defined as:


$$\begin{array}{l} PPV=TP/\left(TP+FP\right) \end{array}$$

and reflects the proportion of relevant detections among all the detected changes.

The TPR is defined as:


$$\begin{array}{l} TPR=TP/\left(TP+FN\right) \end{array}$$

and reflects the proportion of the ground-truth changes that have been detected.

The *local* DSC correspond the DSC computed on a restricted area defined as the dilation with a 4-voxel radius spherical structuring element to the ground truth. This metric enables us to focus the evaluation on the local spatial accuracy of the detection method

In addition to the voxel-wise metrics, we also report lesion-wise metrics, namely the Lesion True Positive Ratio (L-TPR) and the Lesion Positive Predictive Value (L-PPV). These metrics have been evaluated thanks to the *animaSegPerfAnalyzer* validation tool while considering the same hyperparameters as in Commowick et al. (2018).

Since all these metrics are not relevant for data that do not exhibit any changes, we consider in that specific case the number of detected connected components as well as the volume of detected changes to characterize the false positive detections.

#### 5.1.5. Variants used for comparison

To demonstrate the benefits of the proposed joint modeling, we consider three variants of the change detection framework:

- *joint*: the proposed joint change detection and registration method described in Section 3.
- *sequential*: The sequential counterpart of the proposed method, which successively performs deformable registration and change detection. For the two steps, we use the same model and optimization algorithms as in substeps of the *joint* approach described in Section 3.3.
- *affine*: The *sequential* approach where the deformable registration has been replaced by affine registration, which corresponds to the most common case. The affine registration was estimated using ANTs library (Avants et al., 2011) ^3^ with default parameters and mutual information metric. Then, the thresholding and smoothing of the change map routine follows model (Equation 14).

### 5.2. Results

#### 5.2.1. Synthetic dataset

**Figure 2:**
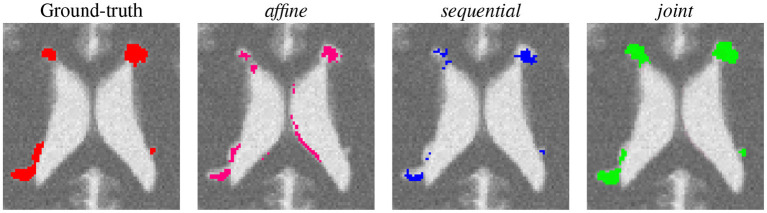
Qualitative comparison of the binary change detection maps obtained with the three methods on the synthetic dataset (lesion appearance with atrophy scenario).

First, a qualitative visual comparison of the three methods is provided in Figure 2 for the lesion appearance with atrophy scenario. The *affine* method succeeds to detect almost all the lesion areas, but it suffers from false positive detection around the ventricles due to brain atrophy. Both the *sequential* and the *joint* methods compensated for brain atrophy deformation since none of them exhibit false detections around the ventricles. However, the *sequential* method failed to detect the whole lesion areas due to the over-compensation of lesion changes. This limitation is overcome by the *joint* approach that succeeds to detect the entire lesion areas.

A quantitative comparison of the three methods under four scenarios is provided in Table 2. First, we consider the appearance of lesion without atrophy. Unsurprisingly, this scenario is the most favorable for the *affine* method since there is no geometric difference to compensate. The *sequential* approach yields to significantly lower values of DSC and local DSC. This is due to the lesion over-compensation effect, as supported by the observed low TPR value (i.e., lack of sensitivity) and high PPV value (i.e., high specificity). Finally, the *joint* approach overcomes the shortcoming of the *sequential* approach and have performances similar to the *affine* method, with a slight tendency to underestimate the detected area. Similar observations can be made about the second scenario involving lesion growth without atrophy.

**Table 2:** Results computed on the synthetic dataset.

**Scenario**	**Method**	**DSC**	**PPV**	**TPR**	**Local DSC**
Lesion appearance no atrophy	*affine*	**0.830**	0.782	**0.885**	**0.830**
	*sequential*	0.684	**0.921**	0.544	0.684
	*joint*	0.814	0.887	0.751	0.814
Lesion growth no atrophy	*affine*	0.766	0.635	**0.964**	0.770
	*sequential*	0.685	**0.734**	0.641	0.688
	*joint*	**0.806**	0.726	0.902	**0.808**
Lesion appearance simulated atrophy	*affine*	0.460	0.329	**0.767**	**0.810**
	*sequential*	0.626	**0.960**	0.465	0.627
	*joint*	**0.743**	0.925	0.621	0.744
Lesion growth simulated atrophy	*affine*	0.652	0.505	**0.919**	0.827
	*sequential*	0.753	**0.869**	0.664	0.754
	*joint*	**0.847**	0.833	0.861	**0.848**

The bold values indicate the highest scores among the four methods.

The conclusions are drastically different for the two scenarios involving simulated atrophy. The performance of the *affine* method is significantly hampered by the numerous false detections due to the atrophy. This is illustrated by the significant decrease of the DSC and PPV values compared to the cases without atrophy, while the TPR and local DSC values are less modified. The *sequential* approach succeeds to compensate for the simulated brain atrophy, as highlighted by the high PPV value, but still underestimates the changes to detect, as indicated by the low TPR value. The *joint* approach clearly outperforms the two previous approaches in terms of detection accuracy, as objectified by the significantly higher DSC value.

**Figure 3:**
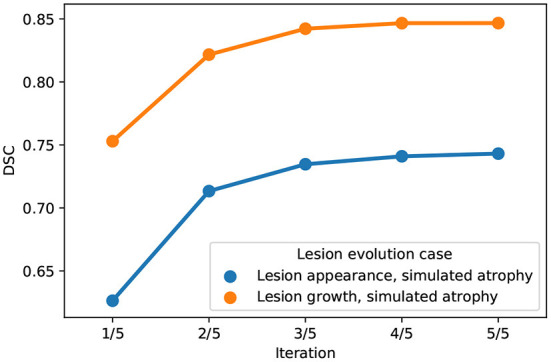
Evolution of DSC across iterations of the alternating optimization scheme of the *joint* approach on the synthetic dataset (blue: Lesion appearance, simulated atrophy, orange: Lesion growth, simulated atrophy).

The behavior of the *joint* approach can be monitored through the iterations of the alternating optimization scheme (see Figure 3). We can see that the DSC increases across the iterations, and the convergence is reached in a few iterations. Concerning the computational burden of the joint approach, it is about 24min on one single core (Intel(R) Xeon(R) Gold 6130 CPU @ 2.10GHz) for an experiment on the synthetic dataset (image size: 181 x 217 x 181).

#### 5.2.2. LesjakDB

**Figure 4:**
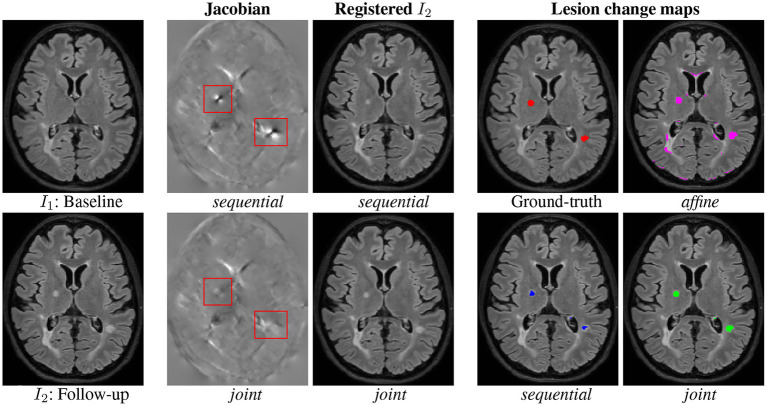
Qualitative comparison of the binary change detection maps obtained with the three methods and of the jacobian of the deformation field estimated with the *sequential* and *joint* approaches on one selected subject from the MSSEG-2 dataset. Hyperparameters: λ_1_ = 70 (for *sequential* and *joint* methods), λ_2_ = 25, λ_3_ = 3. *I*_1_: Baseline, *I*_2_: Follow-up, *sequential, joint*, Ground-truth, and *affine*.

First, a qualitative visual comparison of the three methods is provided in Figure 4. We can draw similar conclusions as for the synthetic dataset (see Figure 2). The *affine* method demonstrates a high sensitivity (i.e., the lesion evolution is well detected) but a lack of specificity (i.e., numerous false positive detections are detected around the ventricles and in the posterior part of the cortex). Conversely, the *sequential* method has high specificity but lacks sensibility. The *joint* approach provides the best visual results, thus illustrating its ability to achieve both high sensitivity and high specificity. Figures 4 show the Jacobian of the deformation fields obtained by the *sequential* and *joint* methods, respectively. The specific pattern characterized by the alternance of both high and low values of the jacobian (see areas highlighted by the red squares in Figure 4) reflects the high local contraction and dilation induced by the deformation field to make the lesion disappear, thus explaining the lack of sensitivity of the detection results.

**Figure 5:**
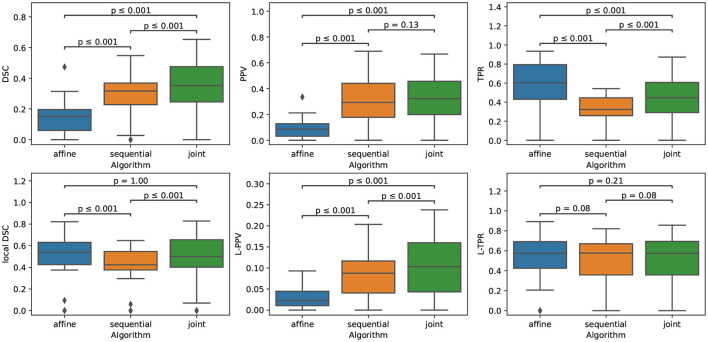
Boxplots corresponding to the results summarized in Table 3 for LesjakDB (*N*_*Subject*_ = 20). Statistical significancy is evaluated thanks to the Wilcoxon signed-rank test between each pair of methods while applying Benjamini/Hochberg FDR correction.

The quantitative evaluation shown in Table 3 (first row) and in Figure 5 confirms the conclusions of the visual analysis. The high sensitivity of the *affine* method is objectified at the voxel level by a statistically significantly higher TPR than the two other methods. At the lesion level, all the three methods exhibit similar L-TPR values, thus emphasizing their ability to detect the same amount of changing areas. Both the *sequential* and *joint* methods yield significantly higher PPV and L-PPV compared to the *affine* method, which illustrates their ability to reduce the number of false detections induced by brain atrophy at both voxel and lesion levels. This result highlights the benefit of using deformable registration in the context of MS lesion monitoring. The significantly lower TPR achieved by the *sequential* method compared to the *joint* method is the consequence of the lesion over-compensation effect. Finally, the *joint* approach significantly outperforms the two other approaches in term of voxel-wise global accuracy (see DSC).

**Table 3:** Results computed on LesjakDB and MSSEG-2-Change datasets.

**DataSet**	**Method**	**Local DSC**	**DSC**	**PPV**	**TPR**	**L-PPV**	**L-TPR**
LesjakDB	*affine*	**0.539 ± 0.174**	0.152 ± 0.087	0.086 ± 0.060	**0.603 ± 0.223**	0.022 ± 0.022	0.576 ± 0.195
	*sequential*	0.424 ± 0.139	0.317 ± 0.117	0.293 ± 0.152	0.323 ± 0.125	0.088 ± 0.051	**0.577 ± 0.197**
	*joint*	0.501 ± 0.179	**0.353 ± 0.144**	**0.323 ± 0.148**	0.447 ± 0.207	**0.103 ± 0.061**	0.574 ± 0.208
MSSEG-2-Change	*affine*	**0.626 ± 0.224**	0.142 ± 0.165	0.081 ± 0.139	**0.633 ± 0.269**	0.015 ± 0.042	0.840 ± 0.271
	*sequential*	0.520 ± 0.196	0.310 ± 0.178	0.298 ± 0.264	0.379 ± 0.176	0.095 ± 0.150	**0.872 ± 0.307**
	*joint*	0.579 ± 0.219	**0.356 ± 0.208**	**0.336 ± 0.254**	0.474 ± 0.222	**0.111 ± 0.155**	**0.872 ± 0.304**
MSSEG-2-Change inverse	*affine*	0.626 ± 0.224	0.142 ± 0.165	0.081 ± 0.139	**0.633 ± 0.269**	0.015 ± 0.042	0.840 ± 0.271
	*sequential*	0.625 ± 0.217	0.348 ± 0.216	0.290 ± 0.244	0.550 ± 0.243	**0.094 ± 0.151**	**0.977 ± 0.292**
	*joint*	**0.655 ± 0.237**	**0.378 ± 0.233**	**0.312 ± 0.250**	0.619 ± 0.266	0.091 ± 0.164	0.947 ± 0.304

The median ± the median absolute deviation (MAD) computed over all subjects are reported for each metric. The MSSEG-2-Change inverse experiment consist in swapping the baseline and follow-up images to evaluate the ability of the methods to detect disappearing lesions. The bold values indicate the highest scores among the three methods.

**Figure 6:**
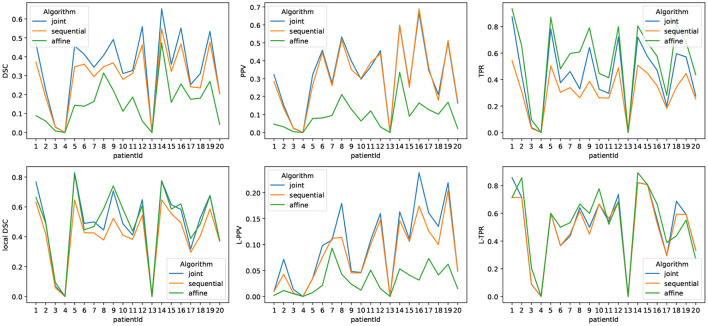
Metrics reporting the performance of the three methods for each subject of LesjakDB.

**Figure 7:**
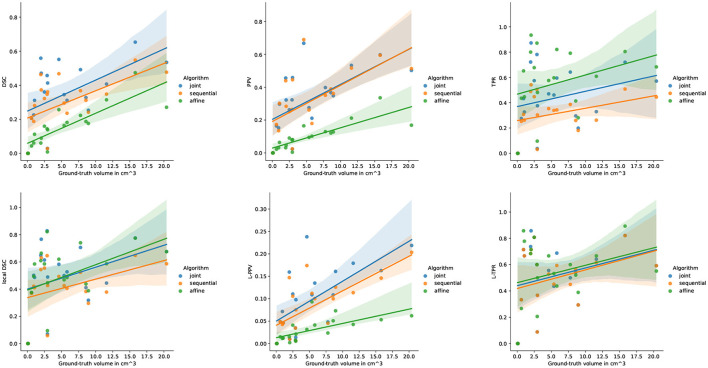
Correlation between the performance of the three methods and the ground-truth evolving lesion load (cm^3^) of the subjects of LesjakDB.

Figure 6 highlights the variability of the performance of the methods across the subjects. It is interesting to notice that, although the performance of the detection methods greatly varies from one subject to the other, the ranking among the three methods appears to be highly consistent across the subjects. When investigating for the factors that may explain the observed variability, it appears that the volume of the ground-truth seems to play a prominent role: the larger is the volume to detect, the better is the performance of the change detection algorithm (see Figure 7).

#### 5.2.3. MSSEG-2

**Figure 8:**
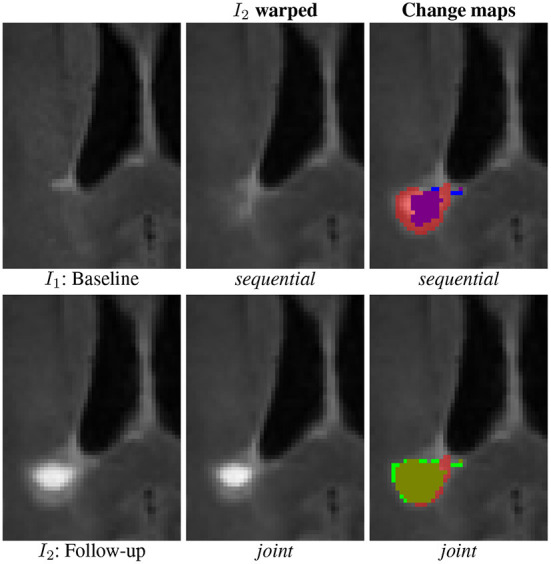
Qualitative comparison of the binary change detection maps obtained with the *sequential* (purple) and *joint* (green) approaches as compared to the ground truth (underlying transparent red) on one selected subject from the MSSEG-2 dataset.

Similarly as for the synthetic and LesjakDB datasets, the qualitative visual comparison of the two approaches based on deformable registration highlights the lack of sensitivity of the *sequential* method due the lesion over-compensation effect (see Figure 8). The resulting change detection map (purple) is too small compared to the ground-truth (underlying transparent red) due to the deformable registration that significantly shrinks the lesion. With the *joint* approach, the shape of the lesion is almost preserved in the warped follow-up image and the change detection map (green) matches almost perfectly the ground-truth.

**Figure 9:**
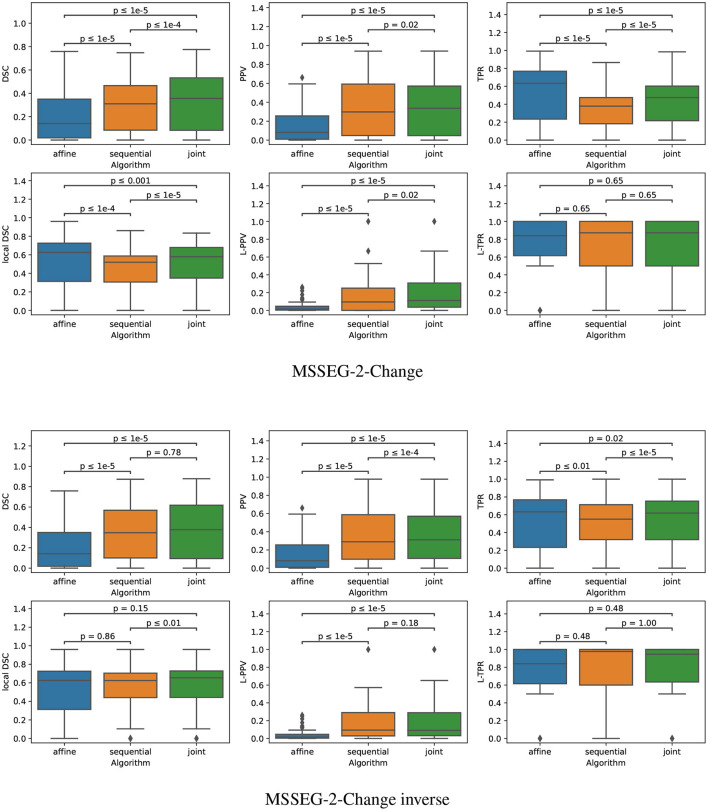
Boxplots corresponding to the results summarized in Table 3 for both MSSEG-2-Change and MSSEG-2-Change inverse (*N*_*Subject*_ = 61). Statistical significancy is evaluated thanks to the Wilcoxon signed-rank test between each pair of methods while applying Benjamini/Hochberg FDR correction.

The quantitative evaluation on the subset MSSEG-2-Change is reported in the second row of Table 3 and in the upper part of Figure 9.

The fact that both the *sequential* and *joint* approaches lead to significantly higher PPV values as compared to the *affine* approach advocates the use of deformable registration to reduce the number of false detections. The benefit of considering the *joint* over the *sequential* approach to overcome the lesion overcompensation effect is clearly demonstrated by the significantly higher TPR and local DSC values obtained with *joint* method.

It is also interesting to notice that the lesion over-compensation effect does not affect the special case of disappearing lesion. Indeed, when registering an image without lesion on a image with a lesion, the dissimilarity in the area of the disappearing lesion cannot be corrected by the transport of intensity of the registration (this is in fact only the case for non symmetric image registration method, see Noblet et al. (2004) for further explanations). To illustrate this phenomenon, we consider the MSSEG-2-Change inverse experiment (see the third row of both Table 3 and the bottom part of Figure 9) that consist in swapping the baseline and the follow-up image, so that the ground-truth now correspond to disappearing lesions. The same conclusion can be drawn from the DSC, PPV, and TPR as compared to the MSSEG-2-Change experiment. The most interesting point concern the local DSC that focuses the evaluation on the disappearing lesion. In that case, there is no significant difference any more between *sequential* and *joint* approaches contrary to the MSSEG-2-Change experiment, showing the absence of lesion over-compensation effect in the specific scenario of detecting disappearing lesions.

**Figure 10:**
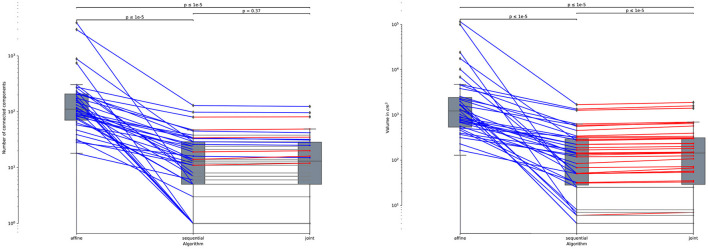
Number of connected components **(left)** and volume in *cm*^3^ **(right)** of the detected changes computed for the 39 subjects of MSSEG-2-NoChange datasets (i.e., subjects without new appearing lesion). Statistical significancy is evaluated thanks to the Wilcoxon signed-rank test between each pair of methods while appling Benjamini/Hochberg FDR correction.

Note that all the results presented above in this section are evaluated on the 61 subjects of MSSEG-2-Change (i.e., subject presenting at least one new appearing lesion). Indeed, the presented metrics cannot be computed anymore for the 39 subjects of MSSEG-2-NoChange since the ground-truth change detection map is empty. This is why we only report the volume of detected changes for this subset of MSSEG-2 (see Figure 10). We can notice that both *sequential* and *joint* approaches lead to significantly lower volume of detected changes as compared to the *affine*, which appears in line with previous findings that support the use of deformable registration to reduce the number of false detections. Also note that the *joint* method yields consistently to slightly higher volume of detected changes as compared to the *sequential* method. This is also the consequence of the lesion over-compensation effect that affects the *sequential* appproach.

## 6. Conclusion and perspectives

We have presented a method that unifies registration and change detection for the analysis of longitudinal brain MRI. It is based on the joint modeling of these two tasks as the minimization of a single objective function, for which we have developed an efficient alternating optimization method. The proposed approach has been evaluated in the context of the follow-up of multiple sclerosis lesion, which requires deformable registration to capture characteristic brain atrophy, but also with the potential caveat to shrink appearing lesions. In this context, the conventional sequential detection pipeline leads to large detection inaccuracies around new appearing lesions. We have demonstrated on simulated and real data that the proposed joint approach is able to combine the ability of deformable registration to correct brain atrophy, and a good preservation of the lesions shape to ensure accurate change detection.

The implementation presented in this paper of the proposed joint model relies in fact on quite simple modeling assumptions. The versatility of the optimization approach opens the way for more sophisticated models that could be handled in the same framework. Alternative data fidelity terms such as cross correlation or mutual information, and regularizers such as total variation could be considered to potentially improve the performance of the method. Another perspective is to improve the convergence of the alternating optimization strategy to ensure a better robustness to local minima. To this end we could consider a fuzzy change detection map to turn its estimation into a continuous optimization problem. This would allow us to use more robust optimization approaches such as Proximal Alternating Linearized Minimization (Bolte et al., 2014). Finally, while we have addressed in this paper the registration problem, the unification principle could be extended to other steps of the change detection pipeline. In particular, the intensity normalization and the bias field inhomogeneity correction of the MRI acquisitions are crucial pre-processing tasks that are impacted by the presence of evolving lesions. Therefore, the integration of these two tasks in a single unified model would be a natural extension of the proposed framework.

## Data availability statement

Publicly available datasets were analyzed in this study. This data can be found at: https://brainweb.bic.mni.mcgill.ca/selection_ms.html; http://lit.fe.uni-lj.si/tools.php?lang=eng; https://portal.fli-iam.irisa.fr/msseg-2/.

## Author contributions

The proposed method was coded by ED. ED, DF, and VN equally contributed to the writing of the article and the interpretation of the results. SK provided a clinical expertise for the analysis of the results and the real annotated databases. All authors contributed to the article and approved the submitted version.

## Footnotes

^1^ https://github.com/ANTsX/ANTs

^2^ https://surfer.nmr.mgh.harvard.edu/fswiki/Samseg

^3^ https://github.com/ANTsX/ANTs

## References

[B1] Altay E. E. Fisher E. Jones S. E. Hara-Cleaver C. Lee J.-C. Rudick R. A. (2013). Reliability of classifying multiple sclerosis disease activity using magnetic resonance imaging in a multiple sclerosis clinic. JAMA Neurol. 70, 338–344. 10.1001/2013.jamaneurol.211 23599930 PMC3792494

[B2] Avants B. Epstein C. Grossman M. Gee J. (2008). Symmetric diffeomorphic image registration with cross-correlation: evaluating automated labeling of elderly and neurodegenerative brain. Med. Image Analysis 12, 26–41. 10.1016/j.media.2007.06.004 17659998 PMC2276735

[B3] Avants B. B. Tustison N. J. Song G. Cook P. A. Klein A. Gee J. C. (2011). A reproducible evaluation of ANTs similarity metric performance in brain image registration. Neuroimage 54, 2033–2044. 10.1016/j.neuroimage.2010.09.025 20851191 PMC3065962

[B4] Bolte J. Sabach S. Teboulle M. (2014). Proximal alternating linearized minimization for nonconvex and nonsmooth problems. Math. Program. 146, 459–494. 10.1007/s10107-013-0701-929993419

[B5] Bosc M. Heitz F. Armspach J.-P. Namer I. Gounot D. Rumbach L. (2003). Automatic change detection in multimodal serial MRI: application to multiple sclerosis lesion evolution. Neuroimage 20, 643–656. 10.1016/S1053-8119(03)00406-3 14568441

[B6] Bostan E. Lefkimmiatis S. Vardoulis O. Stergiopulos N. Unser M. (2014). Improved variational denoising of flow fields with application to phase-contrast MRI data. IEEE Signal Process. Lett. 22, 762–766. 10.1109/LSP.2014.2369212

[B7] Boyd S. Parikh N. Chu E. Peleato B. Eckstein J.. (2011). Distributed optimization and statistical learning via the alternating direction method of multipliers. Foundat. Trends Mach. Learn. 3, 1–122. 10.1561/2200000016

[B8] Boykov Y. Veksler O. Zabih R. (2001). Fast approximate energy minimization via graph cuts. IEEE Trans. Pattern Anal. Mach. Intell. 23, 1222–1239. 10.1109/34.96911426441457

[B9] Bruhn A. Weickert J. Schnörr C. (2005). Lucas/kanade meets horn/schunck: combining local and global optic flow methods. Int. J. Comput. Vis. 61, 1–21. 10.1023/B:VISI.0000045324.43199.43

[B10] Cabezas M. Corral J. Oliver A. Díez Y. Tintor,é M. Auger C.. (2016). Improved automatic detection of new T2 lesions in multiple sclerosis using deformation fields. Am. J. Neuroradiol. 37, 1816–1823. 10.3174/ajnr.A4829 27282863 PMC7960461

[B11] Cerri S. Puonti O. Meier D. S. Wuerfel J. Mühlau M. Siebner H. R.. (2021). A contrast-adaptive method for simultaneous whole-brain and lesion segmentation in multiple sclerosis. Neuroimage 225, 117471. 10.1016/j.neuroimage.2020.117471 33099007 PMC7856304

[B12] Cocosco C. A. Kollokian V. Kwan R. K.-S. Pike G. B. Evans A. C. (1997). Brainweb: online interface to a 3D mri simulated brain database. Neuroimage 5, 425.

[B13] COM (2021). MSSEG-2 Challenge Proceedings: Multiple Sclerosis New Lesions Segmentation Challenge Using a Data Management and Processing Infrastructure, COM: Strasbourg.

[B14] Commowick O. Istace A. Kain M. Laurent B. Leray F. Simon M.. (2018). Objective evaluation of multiple sclerosis lesion segmentation using a data management and processing infrastructure. Sci. Rep. 8, 13650. 10.1038/s41598-018-31911-7 30209345 PMC6135867

[B15] Dufresne E. Fortun D. Kumar B. Kremer S. Noblet V. (2020). Joint registration and change detection in longitudinal brain MRI, in 2020 IEEE 17th International Symposium on Biomedical Imaging (ISBI) (Iowa City, IA: IEEE).

[B16] Elliott C. Arnold D. L. Collins D. L. Arbel T. (2013). Temporally consistent probabilistic detection of new multiple sclerosis lesions in brain MRI. IEEE Trans. Med. Imaging 32, 1490–1503. 10.1109/TMI.2013.2258403 23613032

[B17] Fortun D. Storath M. Rickert D. Weinmann A. Unser M. (2018). Fast piecewise-affine motion estimation without segmentation. IEEE Trans. Image Process. 27, 5612–5624. 10.1109/TIP.2018.2856399 30040638

[B18] Ganiler O. Oliver A. Diez Y. Freixenet J. Vilanova J. C. Beltran B.. (2014). A subtraction pipeline for automatic detection of new appearing multiple sclerosis lesions in longitudinal studies. Neuroradiology 56, 363–374. 10.1007/s00234-014-1343-1 24590302

[B19] Hill D. L. Batchelor P. G. Holden M. Hawkes D. J. (2001). Medical image registration. Phys. Med. Biol. 46, R1. 10.1088/0031-9155/46/3/201 11277237

[B20] Kaunzner U. W. Gauthier S. A. (2017). MRI in the assessment and monitoring of multiple sclerosis: an update on best practice. Therapeut. Adv. Neurol. Disord. 10, 247–261. 10.1177/1756285617708911 28607577 PMC5453402

[B21] LaMontagne P. J. Benzinger T. L. Morris J. C. Keefe S. Hornbeck R. Xiong C. (2019). OASIS-3: Longitudinal Neuroimaging, Clinical, and Cognitive Dataset for Normal Aging and Alzheimer Disease. Cold Spring Harbor Laboratory Press. Available online at: https://www.medrxiv.org/content/early/2019/12/15/2019.12.13.19014902.full.pdf

[B22] Lesjak Ž, Z. Pernuš F. Likar B. Špiclin I. (2016). Validation of white-matter lesion change detection methods on a novel publicly available MRI image database. Neuroinformatics 14, 403–420. 10.1007/s12021-016-9301-1 27207310

[B23] Lewis E. B. Fox N. C. (2004). Correction of differential intensity inhomogeneity in longitudinal MR images. Neuroimage 23, 75–83. 10.1016/j.neuroimage.2004.04.030 15325354

[B24] Llad,ó X. Ganiler O. Oliver A. Mart,í R. Freixenet J. Valls L.. (2012). Automated detection of multiple sclerosis lesions in serial brain MRI. Neuroradiology 54, 787–807. 10.1007/s00234-011-0992-6 22179659

[B25] McNamara C. Sugrue G. Murray B. MacMahon P. (2017). Current and emerging therapies in multiple sclerosis: implications for the radiologist, part 1—mechanisms, efficacy, and safety. Am. J. Neuroradiol. 38, 1664–1671. 10.3174/ajnr.A5147 28408630 PMC7963700

[B26] Noblet V. Heinrich C. Heitz F. Armspach J.-P. (2004). A topology preserving non-rigid registration method using a symmetric similarity function - application to 3-D brain images, in European Conference on Computer Vision (ECCV) (Prague), 546-557.

[B27] Radke R. Andra S. Al-Kofahi O. Roysam B. (2005). Image change detection algorithms: a systematic survey. IEEE Trans. Image Process. 14, 294–307. 10.1109/TIP.2004.838698 15762326

[B28] Rey D. Subsol G. Delingette H. Ayache N. (2002). Automatic detection and segmentation of evolving processes in 3D medical images: application to multiple sclerosis. Med. Image Anal. 6, 163–179. 10.1016/S1361-8415(02)00056-7 12045002

[B29] Rousseau F. Faisan S. Heitz F. Armspach J.-P. Chevalier Y. Blanc F.. (2007). An a contrario approach for change detection in 3D multimodal images: application to multiple sclerosis in MRI, in 2007 29th Annual International Conference of the IEEE Engineering in Medicine and Biology Society (Lyon: IEEE), 2069–2072. 10.1109/IEMBS.2007.4352728 18002394

[B30] Salem M. Cabezas M. Valverde S. Pareto D. Oliver A. Salvi J.. (2018). A supervised framework with intensity subtraction and deformation field features for the detection of new T2-w lesions in multiple sclerosis. Neuroimage Clin. 17, 607–615. 10.1016/j.nicl.2017.11.015 29234597 PMC5716954

[B31] Shinohara R. T. Sweeney E. M. Goldsmith J. Shiee N. Mateen F. J. Calabresi P. A.. (2014). Statistical normalization techniques for magnetic resonance imaging. Neuroimage Clin. 6, 9–19. 10.1016/j.nicl.2014.08.008 25379412 PMC4215426

[B32] Song S. Zheng Y. He Y. (2017). A review of methods for bias correction in medical images. Biomed. Eng. Rev. 3, 1550. 10.18103/bme.v3i1.1550

[B33] Sotiras A. Davatzikos C. Paragios N. (2013). Deformable medical image registration: a survey. IEEE Trans. Med. Imaging 32, 1153–1190. 10.1109/TMI.2013.2265603 23739795 PMC3745275

[B34] Sweeney E. Shinohara R. Shea C. Reich D. Crainiceanu C. (2013). Automatic lesion incidence estimation and detection in multiple sclerosis using multisequence longitudinal MRI. Am. J. Neuroradiol. 34, 68–73. 10.3174/ajnr.A3172 22766673 PMC3554794

[B35] Tustison N. J. Avants B. B. Cook P. A. Gee J. C. (2010). N4ITK: improved N3 bias correction with robust B-spline approximation. IEEE Trans. Med. Imaging 29, 1310–1320. 10.1109/ISBI.2010.5490078 20378467 PMC3071855

[B36] Vogel C. Roth S. Schindler K. (2013). An evaluation of data costs for optical flow, in DAGM Symposium on Pattern Recognition (Berlin), 343–353.

[B37] Zhang Y. Brady M. Smith S. (2001). Segmentation of brain mr images through a hidden markov random field model and the expectation-maximization algorithm. IEEE Trans. Med. Imaging 20, 45–57. 10.1109/42.906424 11293691

